# Pharmacokinetics of Orally Administered Fluralaner in Alpacas (*Vicugna pacos*) Affected by Sarcoptic Mange

**DOI:** 10.3390/ani16213399

**Published:** 2026-10-28

**Authors:** Federica Di Cesare, Linda Ucci, Petra Cagnardi, Alessia Libera Gazzonis, Sergio Aurelio Zanzani, Radmila Pavlovic, Denise Drago, Roberto Edoardo Villa, Susanna Draghi, Francesco Arioli, Giulia Sala

**Affiliations:** 1 Department of Veterinary Medicine and Animal Sciences, University of Milan, Via dell’Università 6, 26900 Lodi, LO, Italy; federica.dicesare@unimi.it (F.D.C.); linda.ucci@unimi.it (L.U.); alessia.gazzonis@unimi.it (A.L.G.); sergio.zanzani@unimi.it (S.A.Z.); roberto.villa@unimi.it (R.E.V.); susanna.draghi@unimi.it (S.D.); francesco.arioli@unimi.it (F.A.); giulia.sala1@unimi.it (G.S.); 2 Proteomics and Metabolomics Facility (ProMeFa), IRCCS San Raffaele Scientific Institute, Via Olgettina 60, 20132 Milan, MI, Italy; pavlovic.radmila@hsr.it (R.P.); drago.denise@hsr.it (D.D.)

**Keywords:** alpacas, ectoparasiticides, fluralaner, isoxazolines, mange, pharmacokinetics, *Sarcoptes scabiei*, South American camelids

## Abstract

Sarcoptic mange may cause severe clinical disease in alpacas (*Vicugna pacos*), and treatment outcomes with commonly used acaricides can be variable. Fluralaner, an isoxazoline drug licensed as an insecticide and acaricide in different species, has been reported as a promising off-label option in alpacas, but pharmacokinetic data and evidence from herd settings are lacking. This study aims to define the pharmacokinetic profile of orally administered fluralaner in alpacas naturally affected by sarcoptic mange. A herd of seven adult female alpacas with clinical mange received a single oral dose of fluralaner at 5.2 mg/kg. Serial blood sampling from pre-treatment to 60 days post-administration was performed; then, serum fluralaner concentrations were quantified by a validated UPLC–HRMS-MS/MS method and analyzed using a non-compartmental approach for pharmacokinetic profile determination. Fluralaner showed prolonged systemic exposure, although marked inter-individual variability was observed. Mean C_max_ was 498.18 ± 302.02 ng/mL, reached at 1.6 ± 0.5 days, with an AUC_last_ of 3323.19 ± 1866.60 ng × day/mL and an elimination half-life of 6.8 ± 2.8 days. Quantifiable concentrations persisted for 35.0 ± 11.2 days on average. No adverse effects were observed, and all animals showed progressive clinical improvement. The findings indicate that a single oral dose of fluralaner provides sustained systemic exposure in alpacas, while further studies are warranted to refine the dosing regimen and to assess its clinical efficacy under herd conditions.

## Simple Summary

Sarcoptic mange is a contagious skin disease caused by mites that can seriously affect alpacas, leading to itching, skin lesions, discomfort, and reduced welfare. Available treatments may not always be effective or easy to manage at herd level, and information on alternative options in alpacas is limited. This study evaluated whether a single administration of fluralaner, a drug licensed for the control of mites and other external parasites in dogs, cats, and poultry, could be useful in alpacas naturally affected by sarcoptic mange. The drug was given orally to an affected herd, and blood samples from seven adult females were collected over 60 days to assess the duration of fluralaner systemic persistence. The treatment was well tolerated, moreover the results indicated that oral fluralaner can be quantified in alpacas for several weeks. Further studies are needed to evaluate its effectiveness and optimize the dosing regimen.

## 1. Introduction

South American camelids, including alpacas (*Vicugna pacos*), are becoming increasingly popular in Europe, where they are primarily raised for wool production and kept as pet species [1,2]. With their increasing diffusion, attention to species-specific health issues is essential, particularly in the case of dermatological diseases [3]. Although several mite species have been reported in alpacas, sarcoptic mange is considered the most clinically significant, causing intense pruritus and severe skin lesions and, in severe cases, leading to systemic deterioration secondary to reduced feed intake and even death if left untreated [4,5,6,7,8].

Since no drugs are specifically licensed for use in alpacas, sarcoptic mange is commonly managed with off-label medications [9,10]. Available options include topical and injectable agents, such as amitraz and macrocyclic lactones (e.g., ivermectin, moxidectin), used either alone or in combined solutions [3,7,8,9,11]. In addition to these, fluralaner (FLU) has attracted particular interest due to its distinct pharmacological profile, which was recently reported to be effective following oral administration in a single alpaca case report [10].

Fluralaner is an isoxazoline ectoparasiticide that selectively inhibits arthropod γ-aminobutyric acid (GABA)-gated chloride channels with exceptional potency and specificity [12,13,14].

Conventional acaricidal protocols in alpacas are challenging, as they often require repeated administration and may yield inconsistent outcomes. These limitations are partly attributed to the species-specific pharmacokinetic variability and reduced efficacy of topical formulations, whose absorption can be impaired by the dense fleece and the absence of lanolin in alpaca fiber [3,5,15]. As a consequence, infestations may persist, prolonging the disease course and increasing handling-associated stress, a particularly relevant concern in alpacas [2].

In this context, the distinct mechanism of action of FLU, combined with its prolonged systemic persistence, may allow sustained exposure exceeding the life cycle of *Sarcoptes* spp. mites [16,17,18]. Indeed, from a pharmacokinetic perspective, FLU has demonstrated remarkable characteristics in dogs and cats: it is readily absorbed and exhibits a long elimination half-life, a high apparent volume of distribution, and low clearance; all these properties enable prolonged activity against ectoparasites after a single administration [16,17].

For this reason, in addition to the species for which FLU is licensed—dogs and cats, for the treatment of ectoparasitic infestations, and poultry, for the control of *Dermanyssus gallinae*—this molecule has been successfully employed off-label for the treatment of sarcoptic mange in several other species, including free-ranging and captive wildlife, particularly raccoon dogs, wombats, and black bears [16,18,19,20,21,22]. Similar therapeutic responses have also been reported in alpacas, supporting the translational potential of this molecule across species with different management conditions and exposure patterns to the parasite [10].

The literature highlights the lack of efficient and standardized treatment protocols for sarcoptic mange in alpacas, with limited controlled evidence available to guide therapeutic strategies [5,22]; notably, pharmacokinetic data in this species are absent, and effectiveness in controlling herd-level infestations has not been investigated yet.

Hence, the aim of this study was to define the pharmacokinetic profile of FLU following a single oral administration in a herd of alpacas affected by sarcoptic mange.

## 2. Materials and Methods

### 2.1. Animals

After obtaining ethical approval (Organismo Preposto al Benessere Animale, OPBA protocol No. 83_2023) and written informed consent from the owner, seven adult female alpacas (*Vicugna pacos*) were enrolled in the study.

All subjects of the herd reared for fiber production showed clinical signs attributable to sarcoptic mange infestation, including intense pruritus and characteristic crusting skin lesions located in the perineal region, muzzle, and caudal thighs. The diagnosis of sarcoptic mange was confirmed by clinical examination and deep skin scraping. Following diagnosis, all animals were weighed, underwent hematological and biochemical analysis to assess their general health status, and received a single oral administration of FLU.

Specifically, FLU chewable tablets formulated for dogs (112.5 and 250 mg; Bravecto^®^, MSD Animal Health S.r.l., Segrate, Italy) were combined to approximate the target dose of 5 mg/kg administered in the case report as an oral solution [10]. The tablets were ground using a mortar and pestle, suspended in water, and immediately administered orally by a syringe. To minimize drug loss, any residual material remaining in the syringe was rinsed with additional water and fully delivered to the animal. Food and water were not withheld either before or after FLU administration.

### 2.2. Blood Sample Collection

Blood samples (2 mL) for the determination of FLU pharmacokinetic profile were collected in the farm where the animals were housed.

Before FLU administration, a venous catheter was aseptically placed in the right jugular vein and maintained for 48 h to facilitate blood sample collection during the initial phase of the study and to ensure emergency drug administration if needed.

The first blood sample (T0) was collected from each alpaca before drug administration and then at prefixed time points after treatment, namely, 2, 4, 6, 24, and 48 h and 7, 14, 21, 28, 35, 42, and 60 days. Then, all samples were centrifuged (4000× *g*, 10 min) to obtain serum and frozen at −20 °C; subsequent analyses were performed within 21 days.

### 2.3. Sample Preparation

FLU was extracted from alpaca serum by protein precipitation. Briefly, 100 μL of serum was aliquoted into a 1.5 mL microcentrifuge tube; then, 10 μL of internal standard solution (diclofenac, 10,000 ng/mL in acetonitrile, ACN) was added, and the mixture was vortexed for 5 s.

Subsequently, 400 μL of ACN was added, and the samples were vortexed for 1 min to allow protein precipitation and analyte extraction. Following centrifugation (12,500× *g*, 10 min), 450 μL of the supernatant was transferred into a 1.5 mL microcentrifuge tube and injected into the analytical system.

### 2.4. Mass Spectrometry Analysis of FLU in Alpaca Serum

The samples were analyzed by liquid chromatography coupled with high-resolution tandem mass spectrometry (UPLC–HRMS-MS/MS) using an Agilent 1290 Infinity UHPLC system (Agilent Technologies, Santa Clara, CA, USA) coupled to a TripleTOF 5600 mass spectrometer equipped with a DuoSpray ion source (SCIEX, Framingham, MA, USA).

Chromatographic separation was performed by injecting 10 µL of each sample in duplicate onto an Acquity UPLC BEH C18 column (1.7 µm, 2.1 × 100 mm, Waters Corporation, Milford, MA, USA) maintained at 45 °C. The mobile phases consisted of water containing 0.1% formic acid (FA) as mobile phase A and acetonitrile (ACN) containing 0.1% FA as mobile phase B. The flow rate was set at 500 µL/min. The chromatographic gradient started at 20% B and was maintained for 0.20 min; mobile phase B was then increased to 90% at 5.0 min and maintained at 90% until 8.0 min. The initial conditions (20% B) were restored at 8.30 min and maintained until 12.0 min for column re-equilibration.

Mass spectrometric detection was performed in negative ionization mode. The source-dependent parameters were set as follows: Ion Spray Voltage Floating (ISVF), −4500 V; source temperature, 500 °C; curtain gas, 25 arbitrary units; Ion Source Gas 1, 33 psi; and Ion Source Gas 2, 58 psi. The acquisition method consisted of a TOF-MS survey scan followed by two product-ion experiments, targeting FLU and diclofenac (IS), respectively. The TOF-MS survey scan was acquired over an *m*/*z* range of 100–650, with an accumulation time of 0.2500 s, a declustering potential (DP) of −65 V, and a collision energy (CE) of −10 eV.

For MS/MS analysis, FLU was monitored using the precursor ion at *m*/*z* 554.05, whereas IS was monitored at *m*/*z* 294.04. Product-ion spectra were acquired over an *m*/*z* range of 150–650, with an accumulation time of 0.09998 s. For FLU, the compound-dependent parameters were DP −65 V, CE −40 eV, ion release delay (IRD) 67, and ion release width (IRW) 25. For diclofenac, the corresponding parameters were DP −65 V, CE −20 eV, IRD 67, and IRW 25.

FLU, eluting at a retention time (RT) of 4.56 min, was quantified using the precursor ion at *m*/*z* 554.0483 and the specific product ion at *m*/*z* 494.020, corresponding to the MS/MS transition *m*/*z* 554.0483 → 494.020. Diclofenac, with RT at 3.73 min, was quantified using the precursor ion at *m*/*z* 294.0097 and the specific product ion at *m*/*z* 250.018.

### 2.5. Method Validation

The analytical method was intra-laboratory-validated in compliance with the international guidelines [23].

Prior to quantification of FLU, calibration curves were prepared by diluting a FLU stock solution (10 μg/mL) in alpaca blank serum to obtain seven concentration levels ranging from 1 to 800 ng/mL. Linearity of the calibration model was assessed in triplicate (y = 0.0003596x + 0.00693), yielding correlation coefficients (r^2^) higher than 0.99.

Method performance in terms of precision and accuracy was evaluated using blank serum samples fortified with FLU at three concentration levels (5, 50, and 400 ng/mL). Specifically, for each level, intra-day precision was evaluated using six replicate measurements, while inter-day precision was assessed from eighteen measurements performed on different days. Extraction recovery was evaluated by comparing FLU peak areas in serum samples spiked before extraction with those spiked after extraction at the same concentration. Matrix effect was assessed by comparing post-extraction-spiked samples with neat standard solutions. The main validation parameters are reported in Table 1.

The limit of detection (LOD) and limit of quantification (LOQ) were estimated from the low-concentration calibration range (1–20 ng/mL) according to the equations LOD = 3.3σ/S and LOQ = 10σ/S, where σ represents the residual standard deviation of the regression, and S the slope of the calibration curve. The calculated LOD and LOQ were 0.1 and 1.08 ng/mL, respectively. These limits were additionally supported by spiked samples at 0.1 and 1.0 ng/mL: FLU was detectable at 0.1 ng/mL and yielded a reproducible quantitative response at 1.0 ng/mL.

Method specificity was verified by analyzing 20 blank serum samples, which showed no interfering peaks at the retention time of FLU.

Stability was evaluated in five different samples under freeze–thaw, processed-sample, and long-term storage conditions. Long-term stability was assessed after one week and one month at −20 °C. Under all tested conditions, coefficient of variation values remained below 10%, supporting adequate analyte stability during sample handling, processing, and storage.

Samples with concentrations exceeding the upper calibration limit (800 ng/mL) were diluted with blank serum and reanalyzed prior to quantification; then, the final concentrations were calculated by applying the corresponding dilution factor.

### 2.6. Serum Protein Binding Determination

The serum protein binding of FLU in alpacas was determined using a microfiltration-based approach. Five concentration levels were prepared in duplicate (the lowest and highest points of the calibration curve and three intermediate levels).

For each concentration ranging between 5 and 200 ng/mL, samples were prepared in ACN and in blank alpaca serum (500 μL), according to the volume requirements of the microfiltration devices. Samples prepared in ACN were directly injected into the analytical system. Conversely, serum samples were incubated at 37 °C for 20 min and subsequently centrifuged (10,000× *g*, 10 min) with 30,000 Nominal Molecular Weight Limit cut-off ultrafiltration units, according to the manufacturer’s instructions for the microfiltration devices (Amicon Ultrafree MC-0.5 mL, Centrifugal Filter Unit 30 K, Merck Millipore, Milan, Italy). Then, the filtrate containing the free fraction of FLU was obtained and injected into the UPLC system. For each concentration level, the percentage of serum protein binding was calculated by subtracting the mean analytical response (peak area) obtained in serum (free FLU) from that obtained in ACN (total FLU) and multiplying the result by 100. The final protein binding percentage was expressed as the mean value obtained across the five concentration levels.

Potential non-specific binding of FLU to the ultrafiltration membrane was assessed using FLU-containing protein-free solutions filtered under the same experimental conditions, confirming negligible membrane adsorption.

### 2.7. Pharmacokinetic Analysis

Pharmacokinetic parameters of FLU were derived from serum concentration–time data by using Phoenix WinNonlin^®^ version 8.7 (Certara, USA. Inc., Princeton, NJ, USA), which enables both compartmental and non-compartmental analyses. Finally, FLU pharmacokinetic profile following single oral administration was described by classic non-compartmental analysis. Concentrations of FLU below the LOQ were not included in the pharmacokinetic parameter calculation.

The peak concentrations, C_max_, and the time to peak, T_max_, were obtained by visual inspection from the experimentally observed data. The area under the concentration–time curve from administration to the last measurable concentration (AUC) and the area under the first moment curve (AUMC) were calculated using the trapezoidal method. The mean residence time (MRT) was determined by using the equation MRT = AUMC/AUC [24].

The terminal elimination rate constant (λz) was estimated from at least three terminal concentration–time points, selected by visual inspection of the log-linear terminal phase, requiring a negative slope and an r^2^ ≥ 0.85. The elimination half-life (t_½λz_) was calculated as ln2/λz.

## 3. Results

All alpacas successfully completed the study, and none of the animals enrolled showed any signs of either local or systemic adverse reactions throughout the entire observation period. Mean age was 4.9 ± 2.5 years, whereas the mean weight was 69.0 ± 2.2 kg. All animals received two different tablets at different FLU concentrations (112.5 mg and 250 mg), with a resulting administered dose of 5.2 ± 0.2 mg/kg.

**Figure 1:**
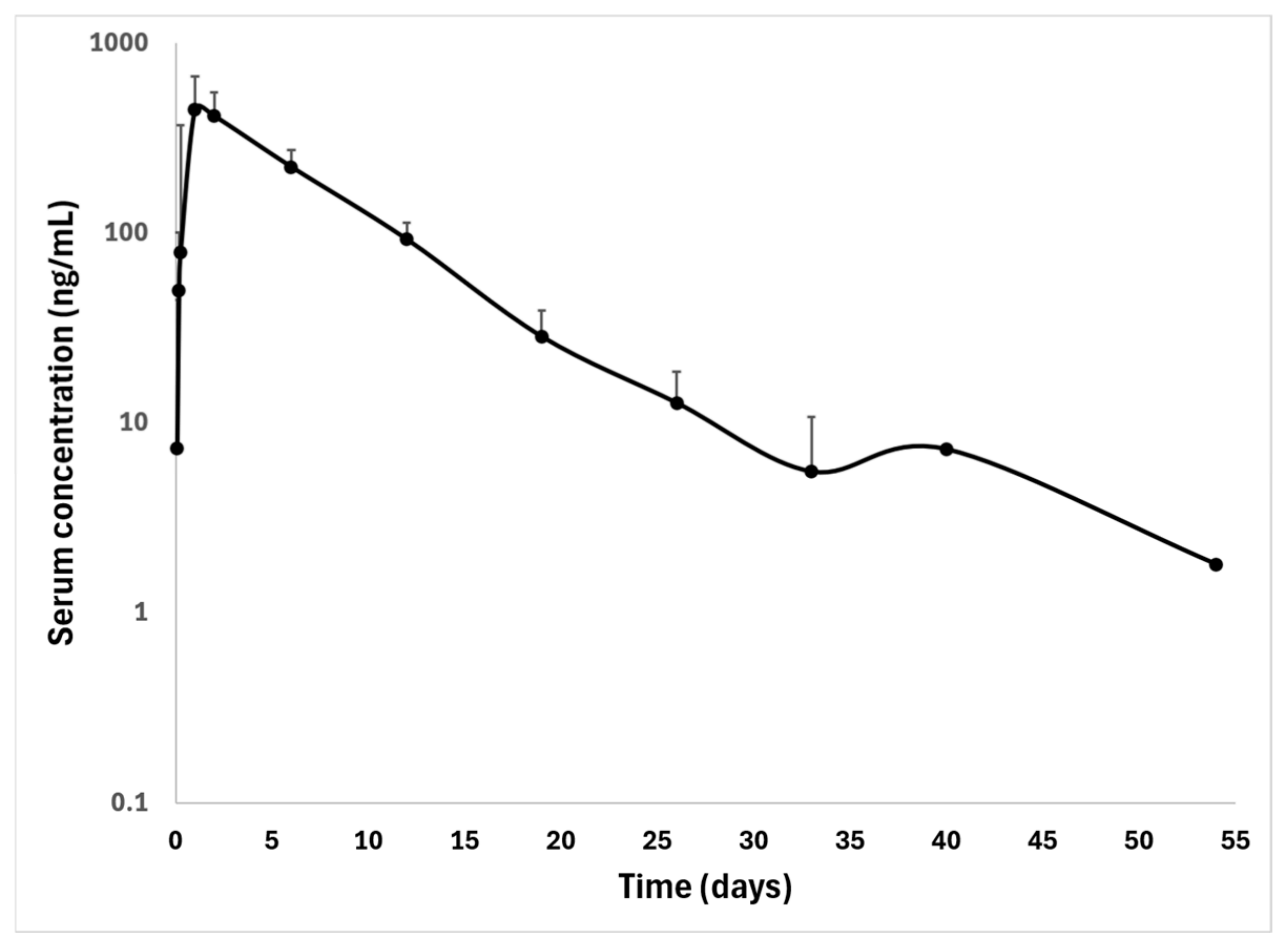
Fluralaner serum concentration + SD following single oral administration at 5.2 ± 0.2 mg/kg in seven alpacas.

Pharmacokinetic parameters data are reported in Table 2 as mean ± standard deviation, except for C_max_, AUC_0–last_, and AUC_0–∞_, for which the geometric mean and coefficient of variation were also calculated. The serum concentration–time profile of FLU is illustrated in Figure 1. Overall, extrapolation beyond the last measurable concentration was minimal, as indicated by an AUC_%Extrap of 1.2 ± 0.7%, supporting the robustness of the exposure estimates.

The protein binding of FLU in alpaca serum was 99.9 ± 0.01%.

## 4. Discussion

In alpacas, sarcoptic mange represents a major health concern because it may cause severe clinical disease, may be fatal if untreated, and can have a marked economic impact due to fleece loss and deterioration in fleece quality [5,6]. Despite its clinical relevance, no drugs are currently licensed for use in this species, and treatment relies largely on off-label administration of available compounds [3,7,8,9,10]. This highlights the need for the development of effective and evidence-based therapeutic protocols, including the evaluation of alternatives such as FLU, already registered for other species [16,18,21]. In this context, the present study describes, for the first time, the pharmacokinetic profile of FLU following oral administration in alpacas.

In the absence of licensed formulations for alpacas, the oral pharmaceutical form was selected based on protocols reported in other species in which FLU is approved, where it has demonstrated high efficacy against mites, including sarcoptic mange [16,21,25]. Although oral formulations are not generally preferred in pseudo-ruminant species, this route was considered the most suitable due to the known limitations of topical administration in alpacas, where drug absorption may be impaired by the absence of lanolin in the fiber [5,26]. Indeed, lanolin is recognized as an excipient supporting topical drug delivery in pharmaceutical formulations and contributes to maintaining skin hydration [27,28,29]. Its absence in dermatological conditions characterized by hyperkeratosis may exacerbate cutaneous dryness and barrier impairment, further limiting drug penetration through the skin [5,26].

Furthermore, at the time of the study, only oral formulations were readily available, and among these options, chewable tablets licensed for dogs were chosen over the solution registered for poultry, as the latter requires specific regulatory compliance (e.g., treatment records and controlled drug storage) that is not applicable to alpaca management systems.

Concerning the administered dose, the choice was based on a previous case report in which one alpaca was successfully treated with a single FLU oral dose at 5 mg/kg (Exzolt^®^, poultry oral solution, MSD Animal Health S.r.l., Segrate, Italy) [10]. The authors selected the dose based on that used in other polygastric species (bighorn sheep, *Ovis canadensis*) [10,25]. However, in the present study, the dose was slightly adjusted because of the indivisible nature of the tablets, and consequently, the animals received a mean dose of 5.2 mg/kg, through the administration of two tablets at different FLU concentrations (112.5 mg and 250 mg). It should be noted that the animals included in the present study were raised on the same farm like the alpaca described in the aforementioned case report [10].

In the evaluation of the pharmacokinetic profile of FLU, three subjects exhibited low C_max_ values (209.60, 289.45 and 278.95 ng/mL), while the other four subjects showed intermediate (437.00 and 432.40 ng/mL) or high values (985.45 and 854.40 ng/mL). This variability may be partly explained by the pseudo-ruminant nature of alpacas, in which differences in forestomach fill at the time of administration can influence drug dilution, retention, and subsequent absorption, ultimately affecting peak plasma concentrations [30,31]. This interpretation is further supported by the observation that, despite the relatively narrow range of administered doses (5.2 ± 0.2 mg/kg), C_max_ varied by almost fivefold among animals, suggesting that factors other than dose, including gastrointestinal conditions at the time of administration, may have contributed substantially to the observed variability in peak exposure.

The T_max_ observed in alpacas (mean 1.6 ± 0.5 days) appears delayed compared with that reported in monogastric species. In dogs, maximum plasma concentrations are typically reached within approximately 1 day following oral administration [16], while even shorter T_max_ values (0.4–0.6 days) have been described in horses [32]. This difference is likely attributable to the pseudo-ruminant digestive physiology of alpacas, as oral drug administration in polygastric species is often associated with pharmacokinetic limitations compared with monogastric animals, due to the complex gastric structure and physiology that can markedly influence drug disposition [30]. In particular, the large volume and compartmentalization of the forestomach may delay gastric emptying, promote drug dilution and binding to digesta, and can expose the compound to microbial degradation prior to absorption, ultimately leading to reduced and more variable bioavailability [30].

However, in the present study, C_max_ and T_max_ may also have been influenced by the method of administration and formulation, as chewing tablets were crushed, mixed, and delivered orally via a syringe, potentially leading to partial drug loss and impaired absorption. Although this approach was preferred from a regulatory perspective, it was not the most practical for administration and may have limited dose standardization.

The elimination half-life (mean 6.8 ± 2.8 days) and the mean residence time (7.0 ± 1.9 days) observed in alpacas were in close agreement, confirming the prolonged systemic persistence of FLU in this species. The elimination half-life appears intermediate compared with that reported in other species: in dogs, FLU shows a markedly longer half-life (approximately 12–15 days), whereas shorter values have been described in horses (approximately 3–6 days) [16,32].

These differences may reflect species-specific variations in drug distribution and metabolism. From a clinical perspective, the persistence of FLU in serum is relevant because sustained systemic exposure is considered important for the successful treatment of sarcoptic mange [33,34]. Given that the life cycle of *Sarcoptes scabiei* lasts approximately 10–14 days, prolonged drug exposure may expose successive generations of mites to the acaricide, including individuals that hatch after treatment [34]. Therefore, although shorter than that reported in dogs, the elimination half-life observed in some alpacas may have allowed systemic exposure to persist across much or all of the parasite life cycle, whereas in others, exposure appeared shorter, highlighting an inter-individual variability that potentially may be relevant to clinical outcome.

**Figure 2:**
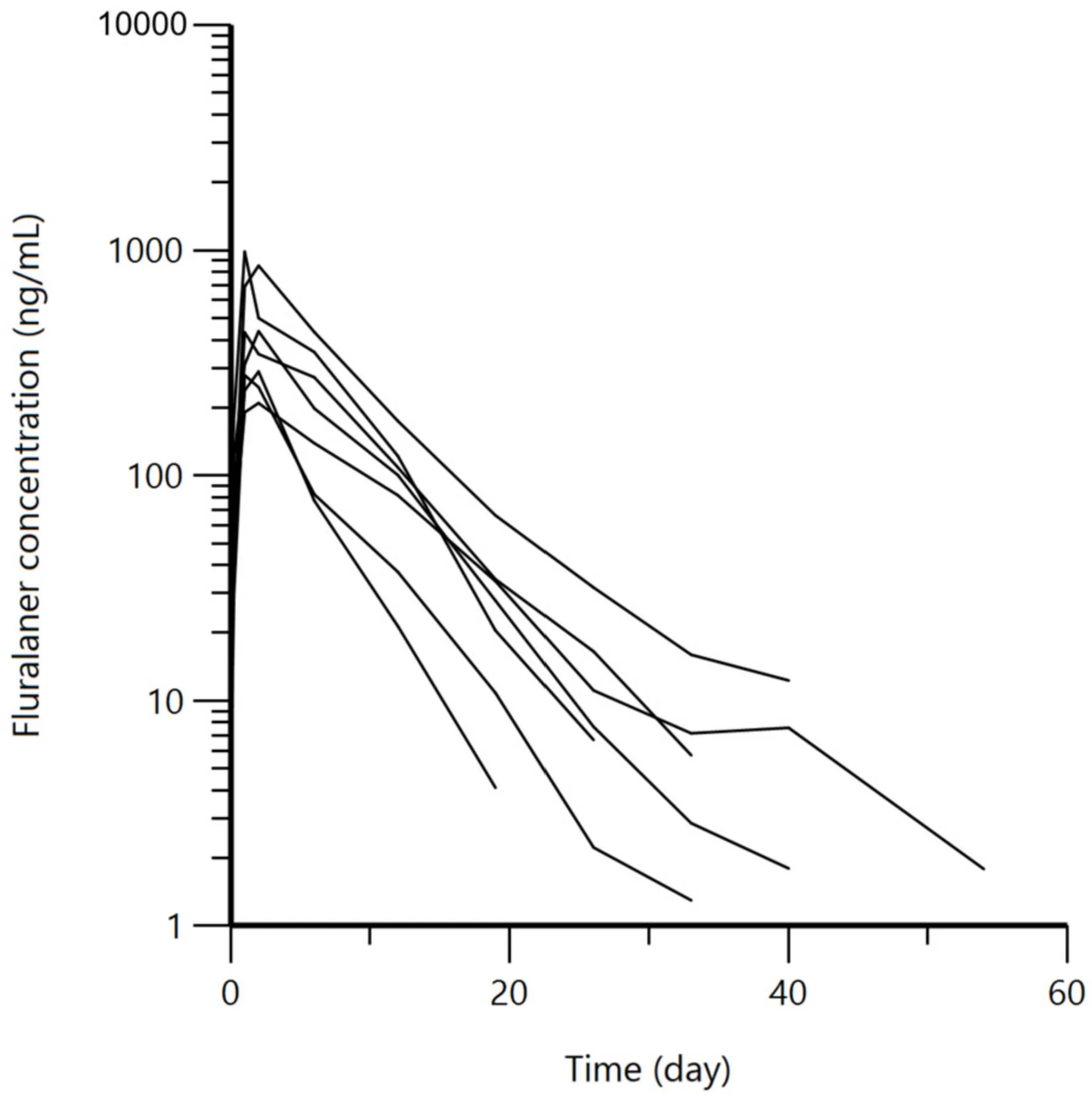
Semi-logarithmic spaghetti plots of the individual disposition curves of fluralaner over 60 days after a single oral administration (5.2 ± 0.2 mg/kg) in seven alpacas.

A marked variability was also detected in both AUC_last_ (3323.19 ± 1866.60 ng × day/mL) and AUMC_last_ (24,010.51 ± 15,217.21 ng × day^2^/mL), indicating substantial differences in systemic exposure and drug exposure over time among animals. In fact, the inspection of the individual concentration–time profiles indicate that variability was not confined to peak exposure (Figure 2). Differences among animals remained evident during the post-absorptive phase, with distinct declines in serum concentrations over time. This is consistent with the dispersion observed for AUC_0–last_ and AUMC_0–last_ and suggests heterogeneity in both the extent and persistence of systemic exposure.

Overall, this variability across pharmacokinetic parameters likely reflects the combined effect of two main factors previously discussed: the pseudo-ruminant gastrointestinal physiology of alpacas, which can influence drug absorption and disposition, and the non-standardized administration method, which may have contributed to variability in dose delivery and oral bioavailability.

At herd-level, such variability may be particularly relevant, as the duration of systemic exposure may differ among individuals and, in some animals, may not extend throughout the entire *Sarcoptes scabiei* life cycle [34]. This variability could reduce the consistency of treatment response, particularly if systemic exposure declines before all parasite developmental stages are affected, potentially allowing persistence of infestation in some animals and subsequent reinfestation within a herd.

In addition to the duration of exposure, the mechanism by which FLU reaches the parasite is also relevant. FLU showed high serum protein binding (approximately 99%), which contributes to its prolonged systemic persistence. In sarcoptic mange, mites burrow into the skin and feed on host-derived fluids, including tissue exudate and capillary leakage associated with skin lesions. This feeding behavior may facilitate the uptake of the active compound, thereby supporting its systemic acaricidal activity [34].

From a clinical perspective, descriptive observations collected during the study indicated rapid improvement following a single oral administration of FLU, including resolution of pruritus and regression of skin lesions, with no apparent adverse effects; however, parasitological recurrence was observed in some alpacas during follow-up.

However, it must be underlined that these observations, which are reported here in the form of descriptive clinical notes, were not part of the present pharmacokinetic study (dedicated manuscript in preparation) and should therefore be interpreted cautiously. Although no conclusions regarding therapeutic efficacy can be drawn from these preliminary observations, this finding may be considered in light of the marked inter-individual pharmacokinetic variability observed in the present study, particularly in systemic exposure (AUC_last_ and AUMC_last_) and terminal elimination profiles. Such variability could potentially result in differences in the magnitude and duration of drug exposure among individuals. Moreover, under field conditions, continued exposure to infested cohabiting animals may further complicate the interpretation of apparent treatment failure or recurrence.

Taken together, the pharmacokinetic findings and these preliminary field observations suggest that the adequacy of a single-dose regimen for the long-term control of sarcoptic mange in alpacas warrants further investigation. In particular, whether repeated administration, potentially timed according to the approximately 10–14-day life cycle of *Sarcoptes scabiei*, could provide more consistent parasite control should be specifically evaluated in controlled clinical and parasitological studies.

This study presents some limitations: first, the relatively small sample-size may limit generalization of the results and does not allow a full characterization of inter-individual variability. Additionally, another limitation is that none of the alpacas enrolled in the present study showed severe clinical disease. Therefore, the pharmacokinetic profile observed here may not fully reflect drug disposition in more severely affected animals, in which systemic inflammation, protein loss, or reduced feed intake could potentially alter fluralaner pharmacokinetics.

Finally, the study design did not include multiple treatment groups, thus precluding direct comparison between different dosing regimens of administration intervals. Accordingly, controlled clinical trials are warranted to investigate and optimize therapeutic protocols, allowing assessment of the relationship between pharmacokinetic parameters, efficacy, and dosing strategies in alpacas.

## 5. Conclusions

In conclusion, this study provides the first characterization of the pharmacokinetic profile of FLU following a single oral administration in alpacas. The prolonged systemic exposure and the high inter-individual variability suggest a heterogeneous disposition of FLU in this species, emphasizing the importance of species-specific pharmacokinetic data when considering its off-label use.

In particular, this variability may be especially relevant under herd conditions and warrants further investigations to define appropriate dosing regimens for sarcoptic mange in alpacas. Future studies should also evaluate alternative routes of administration and newly available formulations to determine whether more predictable systemic exposure can be achieved.

## Figures and Tables

**Table 1:** Intra-laboratory validation parameters of the analytical method for FLU quantification in alpaca serum.

	Concentration Level		
	*Low* *(5 ng/mL)*	*Medium* *(50 ng/mL)*	*High* *(400 ng/mL)*
Accuracy (% Bias)	−8.7	−2.3	4.6
Intra-day precision (% CV) (*n* = 6)	10.4	12.3	9.6
Inter-day precision (% CV) (*n* = 18)	11.6	8.2	10.8
Recovery (% ± SD)	84.5 ± 5.6	88.7 ± 4.3	91.2 ± 3.9
Matrix effect (% ± SD)	94.1 ± 6.8	97.6 ± 5.3	108.1 ± 7.9

CV = coefficient of variation; SD = standard deviation.

**Table 2:** Mean and standard deviation of pharmacokinetic parameters calculated with non-compartmental analysis following single oral administration of FLU at 5.2 ± 0.2 mg/kg in alpacas (*n* = 7).

Parameter	Unit	Mean ± Standard Deviation
C_max_	ng/mL	498.18 ± 302.02
		(429.97; 0.42) *
C_last_	ng/mL	4.82 ± 3.91
T_max_	day	1.6 ± 0.5
T_last_	day	35.0 ± 11.2
t_½λz_	day	6.8 ± 2.8
AUC_0–last_	ng × day/mL	3323.19 ± 1866.60
		(2892.97; 0.06) *
AUC_0–∞_	ng × day/mL	3372.53 ± 1920.07
		(2929.09; 0.06) *
AUMC_0–last_	ng × day^2^/mL	24,010.51 ± 15,217.21
AUMC_0-∞_	ng × day^2^/mL	26,442.05 ± 18,421.18
MRT_0–last_	day	7.0 ± 1.9
MRT_0–∞_	day	7.5 ± 2.1
Cl_F	mL/day/kg	2044.9 ± 1151.0
Vz_F	mL/kg	18,705.9 ± 11,081.9

* Geometric mean and coefficient of variation expressed as percentage. C_max_ = maximum concentration observed; C_last_ = last concentration observed; T_max_ = observed time for C_max_; T_last_ = observed time for C_last_; t_½λz_ = elimination half-time; AUC_0–last_ = area under serum concentration–time curve from 0 to last concentration; AUC_0–∞_ = area under serum concentration–time curve from 0 to infinity; AUMC_0–last_ = area under moment curve from 0 to last concentration; AUMC_0–∞_ = area under moment curve from 0 to infinity; MRT_0-last_ = mean residence time from 0 to last concentration; MRT_0–∞_ = mean residence time from 0 to infinity; Cl_F = apparent clearance after oral administration; Vz_F = apparent volume of distribution during the terminal phase after oral administration.

## Data Availability

The data presented in this study are available on request from the corresponding author.

## Abbreviations

The following abbreviations are used in this manuscript:

ACN	Acetonitrile
AUC_0–last_	Area under the serum concentration–time curve from time zero to the last measurable concentration
AUC_0–∞_	Area under the serum concentration–time curve from time zero to infinity
AUMC_0–last_	Area under the first moment curve from time zero to the last measurable concentration
AUMC_0–∞_	Area under the first moment curve from time zero to infinity
CE	Collision energy
Cl_F	Apparent clearance after oral administration
C_max_	Maximum observed concentration
C_last_	Last observed concentration
DP	Declustering potential
FA	Formic acid
FLU	Fluralaner
GABA	γ-Aminobutyric acid
IRD	Ion release delay
IRW	Ion release width
IS	Internal standard
ISVF	Ion Spray Voltage Floating
LOD	Limit of detection
LOQ	Limit of quantification
MRT_0–last_	Mean residence time from time zero to the last measurable concentration
MRT_0–∞_	Mean residence time from time zero to infinity
MS/MS	Tandem mass spectrometry
OPBA	Organismo Preposto al Benessere Animale
RT	Retention time
SD	Standard deviation
T0	Pre-treatment time point
t_½λz_	Terminal elimination half-life
T_max_	Time to maximum observed concentration
T_last_	Time of the last observed concentration
TOF-MS	Time-of-flight mass spectrometry
UHPLC	Ultra-high-performance liquid chromatography
UPLC	Ultra-performance liquid chromatography
UPLC–HRMS-MS/MS	Ultra-performance liquid chromatography coupled with high-resolution tandem mass spectrometry
Vz_F	Apparent volume of distribution during the terminal phase after oral administration
